# The Patterns and Predictors of Loneliness for the Chinese Medical Students Since Post-Lockdown to New Normal With COVID-19

**DOI:** 10.3389/fpubh.2021.679178

**Published:** 2021-07-01

**Authors:** Hui Zhang, Jun Yang, Yuxin Li, Gaoyue Ren, Lina Mu, Yunjiang Cai, Qiusha Luo, Yuqiu Zhou

**Affiliations:** School of Nursing, Harbin Medical University, Harbin, China

**Keywords:** loneliness, patterns, predictors, medical students, COVID-19, latent profile analysis

## Abstract

**Background:** The coronavirus disease 2019 (COVID-19)-related quarantine has had unique psychological challenges for medical students, particularly loneliness. In this study, we demonstrated the patterns and predictors of loneliness in medical students since post-lockdown to new normal with COVID-19.

**Methods:** A convenience sampling method was used in this study. Face-to-face online questionnaires of UCLA Loneliness Scale and psychological characteristics scales were completed by 1,478 participants. Latent profile analysis and multinominal logistic regressions were performed.

**Results:** Three latent profile models were identified in this study: low loneliness (52.3%), interpersonal sensitivity loneliness (3.5%), and high loneliness (44.1%). Sophomore (Est = 1.937; *p* < 0.05) and junior students (Est = 2.939; *p* < 0.05), neuroticism (Est = 2.475; *p* < 0.05), high arousal symptoms (Est = 2.618; *p* < 0.01), and the quality of support from friends (Est = 2.264; *p* < 0.05) were the risk factors for high loneliness profile. In addition, sophomore (Est = 2.065; *p* < 0.05) and junior students (Est = 2.702; *p* < 0.01), openness (Est = 2.303; *p* < 0.05), and conscientiousness personality (Est = −2.348; *p* < 0.05) were the predictors of an interpersonal sensitive loneliness profile. Good peer relationship (Est = −2.266; *p* < 0.05) and other support (Est = −2.247; *p* < 0.05) were protective factors for low loneliness profile.

**Limitations:** Participants were selected from one medical university; the generalizability is limited.

**Conclusions:** Timely loneliness-focused interventions should be targeted on the different profiles and predictors of loneliness in medical students.

## Introduction

The coronavirus disease 2019 (COVID-19) outbreak was at the end of 2019 and had spread immediately all over the world. The global pandemic of COVID-19 has caused multidimensional damages. Evidence of mental health distress associated with the COVID-19 pandemic has been reported globally (1, 2). Several studies have been conducted since the beginning of the pandemic on the psychological well-being of individuals, showing high levels of psychological distress in terms of anxiety, depression, stress, and even PTSD symptomatology (3–6).

Medical students are vulnerable to the COVID-19-related mental health problems because of a number of challenges, including campus closures, less entertainment and interpersonal interaction, stressful academic pressure, delayed internship opportunities and job, reduced access to mental health services, and the future professional acceptance and safety concern after graduation under the effect of pandemic (7, 8). The United States reported that the pandemic had moderate effects on stress and anxiety levels in medical students; 84.1% of students felt at least some anxiety (9). In China, the detection rate of anxiety and depression symptoms was 34.4 and 43.3%, respectively, in the exposed medical students during the pandemic; the same was significantly higher than that before the COVID-19 outbreak (10). Previous study has cautioned that the effects of pandemic-related mental health can exist persistently even after the pandemic ends (11). A longitudinal study on college students has shown V-shaped growth trajectories for stress, anxiety, and depression wherein these variables decreased during lockdown and increased in the post-lockdown period (12). Some studies revealed that college students showed a surge of depression that increased compared with that before the COVID-19 pandemic, whereas anxiety levels declined (13, 14). Social distancing measures had successfully contributed to slowing down the spread of the COVID-19 infection and relieved the public health systems. Additional measures included curfews and closing entertainment stores, schools, and universities, which also continued after the lockdown. Because of situational risk factors associated with the social isolation of medical students (e.g., closed campus, amount and type of social contact, and reduced emotional support), loneliness was suspected as a prominent factor of depression during the pandemic, which could persist for a longer time (15).

Loneliness is the feeling and thought of being isolated and disconnected from others (16). It occurs when individuals perceive their social relationships as insufficient or unsatisfying. Loneliness is associated with social and interpersonal variables (stress, social support, peer relationship, and life event) (17–19) and individual's psychological characteristic (personality and coping style) (20, 21). Evidence shows an increase in loneliness in medical students who were identified as a high-risk population being affected by the pandemic (22). Researchers suggested that loneliness leads to depression and can predict future psychiatric symptoms and physical disorders (23–25). Thus, identifying the marked increase in loneliness in the medical student population can mitigate huge downstream negative effects (26).

To reduce the outbreak of depression and keep sound mental and physical health after pandemic, it is necessary to understand how loneliness manifests and what are its predictors in the medical students, which are of considerable use to the mental health profession and medical educators who can target the high-risk population to take preventable intervention. But little is known about the subtype of loneliness in medical students and whether different groups of individuals can be identified on the basis of their loneliness characteristics. Previously, various methods were used to measure loneliness (single-item vs. multiple-item scales) and to classify individuals as “being alone” by a certain response option for a single-item measure or cut-off score for multi-item scales; these were likely to be biased for loneliness (27–29). However, an assumption that loneliness is a unidimensional construct exists, which was challenged by researchers who then argued that multiple types of loneliness might exist (27). Hawkins-Elder et al. conducted a latent profile analysis (LPA) on 18,264 participants in New Zealand and identified four distinct loneliness profiles: high-loneliness, low-loneliness, appreciated outsiders, and superficially connected (30). Meanwhile, four subtypes of loneliness were classified in the US adults and different types of loneliness have unique associations with mental health status (27). However, the subtypes of loneliness in medical students were lacked. To fill this gap, LPA was performed in this study to identify homogeneous subgroups, or profiles, of individuals in the sample characterized by similar profiles of revised-UCLA (R-UCLA) scores in medical students. Exploring the demographic and psychosocial characteristics that could predict loneliness identified profiles of medical students since post-lockdown to new normal with COVID-19. This results may help the college educators and mental professors to identify the characteristic of the high risk of loneliness in medical students and timely loneliness-focused interventions should be taken. A hypothesis that the sample would comprise several profiles/classes and these profiles would differ quantitatively in relation to R-UCLA scores was considered (e.g., whether participants with poor peer relationship or social support were overrepresented in a possible “high loneliness” profile).

## Materials and Methods

### Participants

A convenience sampling was used, and participants were recruited from Harbin Medical University (Daqing) in Heilongjiang province, which is located in the northeast of China. Data were collected between September and October 2020 (the new normal for COVID-19 that the students were limited to outside of campus). The inclusion criteria were (1) full-time undergraduate or college student, (2) ≥16 years old, and (3) voluntary to participate the survey. The exclusion criteria were (1) students who were in the clinical routine and lived outside of the campus, (2) the students had serious mental disease (schizophrenia, major depressive disorder, bipolar disorder) screened using a mental health survey at the beginning of the semester and diagnosed by the psychiatrist, and (3) incomplete questionnaires (≥20% missing data). A total of 1,492 medical students were invited, of which 10 returned incomplete questionnaires and 4 had serious mental disease who were then excluded. The final data analyses included 1,478 medical students, 1,073 (72.6%) females and 405 (27.4%) males. The mean age was 19.08 years [standard deviation (SD) = 1.16]. There were 687 (46.5%) freshmen, 416 (28.1%) sophomore students, and 375 (27.5%) junior students in this study.

### Procedure

The design of this study was approved by the ethics committee of Harbin Medical University (Daqing). All the participants were invited to their classroom during self-study at night or free time through a notice on the campus publicity board. The first researcher, HZ, explained the aim of this survey and then obtained verbal and electronical signature written consent from those who were willing to participate in the study. The students were asked to complete online questionnaires through the Wechat using their cell phones, which lasted ~20 min. Participants would get ¥5 yuan after the survey.

### Measures

#### Demographic Characteristics

Information on age, gender, grade, place of residence, family status, family income, and the number of siblings was obtained.

#### Revised UCLA Loneliness Scale

Loneliness was assessed with the R-UCLA loneliness scale (16). This is a 20-item self-report four-point Likert scale (1 = never, 4 = often), with the total score ranging from 20 to 80. Higher scores indicated greater loneliness. The internal consistency of the Chinese version was 0.90 (31), and the Cronbach's α was 0.915 in this study.

#### Perceived Stress Scale

Perceived Stress Scale (PSS) is a 14-item self-report scale developed by Cohen to measure stress (32). Each item was recorded on a five-point Likert scale (1 = never, 5 = always), with a total score ranging from 14 to 70; the higher the score, the greater the stress that the individual perceived. The Chinese version of PSS was translated by Yang and demonstrated acceptable reliability and validity (33); the Cronbach's α was 0.78. The Cronbach's α was 0.876 in this study.

#### Revision of Life Event Scale (IES-R)

It is a self-report scale to measure and assess an individual's catastrophic experience of a particular life event. This scale has 22 items and covers three subscales: aggressive symptoms, avoidance symptoms, and high arousal symptoms. Each item was scored on a five-point Likert scale (0 = never, 4 = always), with a total score ranging from 0 to 88 (34). The Cronbach's α of the three subscales ranged from 0.89 to 0.94. The internal consistency of the Chinese version was 0.89 (35). In this study, the Cronbach's α of the whole scale was 0.948, and the Cronbach's α of subscale ranged from 0.869 to 0.896.

#### Simplified Coping Style Questionnaire

The Simplified Coping Style Questionnaire designed by Xie was used to evaluate the coping characteristics in different populations (36). It is a 20-item self-report questionnaire; each item was scored on a four-point Likert scale (0 = do not take, 3 = often take). It contains positive coping and negative coping subscales and demonstrates satisfactory levels of reliability and validity in the Chinese population; the Cronbach's α was 0.90 (36). In this study, the Cronbach's α was 0.829 for the whole scale; the positive and negative coping subscales were 0.881 and 0.759, respectively.

#### Perceived social support scale

The perceived social support scale is a 12-item self-report, seven-point Likert scale (1=extremely disagree, 7=extremely agree) to measure the degree to which individuals perceive support from various sources of social support (37). It includes family support, friend support, and other support subscales. The total score ranged from 12 to 84; the higher the score, the better the social support. The Cronbach's α of the Chinese version was 0.88 (38). The Cronbach's α for the whole scale was 0.952; the Cronbach's α of the subscales ranged from 0.901 to 0.928 in this study.

#### Chinese Big Five Personality Inventory Brief Version

It is a self-report scale developed by Wang and Dai on the basis of the Chinese Big Five Personality Inventory (CBF-PI) (39). It contains 40 items and five subscales: neuroticism, conscientiousness, agreeableness, openness, and extraversion. Each item was scored on a six-point Likert scale (1 = completely out of line, 6 = be completely in conformity). Each subscale of CBF-PI brief version showed acceptable reliability and validity in Chinese college students (40). In this study, the Cronbach's α was 0.84 for the whole scale, and the Cronbach's α for the subscales ranged from 0.802 to 0.883.

#### Peer Relationships Scale

The peer relationship scale developed by Zou investigates individual's perception of their peer relationship (41). It consists of 30 items and two subscales: peer acceptance subscale and peer fear subscale. Each item was scored on a four-point Likert scale (1 = completely disagrees, 4 = completely agrees). The higher the score of peer acceptance, the better the peer relationship. The higher the score of peer fear, the worse the peer relationship. The Cronbach's α of the whole scale was 0.660, and the subscale of peer acceptance and peer fear and inferiority was 0.918 and 0.893, respectively, in adolescence (41). In this study, the Cronbach's α of the whole scale was 0.752, and that of the subscale ranged from 0.944 to 0.933.

#### Patient Health Questionnaire-9

Patient Health Questionnaire-9 developed by Robert Spitzer is based on the primary care mental illness assessment tool, and it is typically used in the diagnosis of clinical depression and screening of the general population (42). It contains nine items, and each item was scored on a four-point Likert scale (0 = not at all, 3 = nearly every day), with a total score ranging from 0 to 27. The cut-off point is 10 in the Chinese population. The Cronbach's α of the Chinese version was 0.85 (43). In this study, the Cronbach's α was 0.905.

### Statistical Analysis

Descriptive statistics and frequency analysis of demographic characteristics were analyzed using SPSS (Version 22.0, SPSS Inc., Chicago, USA). Independent *t*-tests and analysis of variance (ANOVA) analyses were performed to investigate the differences in loneliness and psychological characteristics between gender and grades.

Mplus (7.4, Muthen & Muthen, Los Angeles, USA) was used to perform the LPA. LPA was performed to identify patterns of loneliness in medical students. LPA is a statistical method that explains the correlation between explicit indicators by means of intermittent latent variables. LPA could group individuals into different categories according to the probability distribution of various responses of explicit variables, and individuals similar to each other were included in one category and those different from other individuals in another (44). Model ML was used to analyze data (45). We tested 1–5 different models, with 1 used as the baseline profile model. Model fit was evaluated using Akaike information criterion (AIC), Bayesian inform criteria (BIC), aBIC, entropy, Lo–Mendell–Rubin likelihood ratio test (LMR), and Bootstrap likelihood ratio test (BLRT). The lower AIC and BIC values were considered to provide a better fit to the data, and a *p*-value < 0.05 for LMR and BLRT indicated that the model with one less class should be rejected in favor of the estimated model. Entropy is a measure of classification accuracy with values ranging from 0 to 1, the higher values indicating greater precision (46).

Multinominal logistic regressions were performed to test the predictors (demographic, stress, coping, social support, life event, personality, peer relationship, and depression) of different profile class of loneliness.

## Results

### Preliminary Results

The demographic characteristics of participants are presented in Table 1. Table 2 shows the comparison results of psychosocial variables between gender and grades. Independent *t*-test showed that there were significant gender differences in loneliness, stress, social support, neuroticism, conscientiousness, agreeableness, openness, and peer fear (*p* < 0.05). The ANOVA results showed that grades were different for loneliness, stress, life event, coping style, social support, neuroticism, peer acceptance, and peer fear (*p* < 0.05). The *post-hoc* analyses revealed that freshmen reported the highest level of loneliness, perceived stress, life event, negative coping, social support, neuroticism, and peer fear among the three grades. Sophomore medical students had the highest level of positive coping and peer acceptance.

**Table 1:** Demographic of medical students (*N* = 1,478).

		***N* = 1,478 (*N*%) M (SD)**
Gender	Male	1,073 (72.6%)
	Female	405 (27.4%)
Age		19.08 (1.16)
Grade	Freshman	687 (46.5%)
	Sophomore	416 (28.1%)
	Junior	375 (25.4%)
Place of residence	Reside1-City	854 (57.8%)
	Reside2-Town	373 (25.2%)
	Reside3-Village	251 (17.0%)
Family status	Nuclear family	955 (64.6%)
	Single parent family	148 (10.0%)
	Joint family	375 (25.4%)
Family income (RMB)	<3,500 Yuan	532 (36.0%)
	3,500–5,000 Yuan	514 (34.8%)
	5,001–8,000 Yuan	274 (18.5%)
	>8,000 Yuan	158 (10.7%)
Number of siblings	0	741 (50.1%)
	1	475 (32.1%)
	2	183 (12.4%)
	≥3	79 (5.4%)

**Table 2:** The differences of loneliness and psychosocial variables between genders and grades.

**Variables**	**Gender**					**Grade**							
	**Boys (*****n*** **=** **405)**		**Girls (*****n*** **=** **1,073)**			**Freshman**		**Sophomore**		**Junior**			
	**M**	**SD**	**M**	**SD**	***t***	**M**	**SD**	**M**	**SD**	**M**	**SD**	***F***	***post-hoc***
Loneliness	38.24	10.45	39.64	10.45	2.300^*^	40.74	10.59	38.18	10.43	37.74	9.94	13.264^**^	1 > 2 > 3
Stress (PSS)	35.14	9.23	36.74	8.99	3.024^**^	37.95	9.36	34.85	8.78	34.90	8.38	21.664^**^	1 > 3 > 2
Life event (IES-R)	24.69	17.61	24.63	16.28	−0.059	28.08	16.51	22.57	15.66	20.67	16.75	29.64^**^	1 > 2 > 3
Coping style (SCSQ)													
Positive coping	23.21	7.14	23.42	6.73	0.518	22.62	6.38	24.72	6.48	23.21	7.78	12.61^**^	2 > 3 > 1
Negative coping	9.50	4.78	9.65	4.42	0.567	9.89	4.42	9.57	4.40	9.12	4.80	3.60^*^	1 > 2 > 3
Social support (PSSS)	61.10	14.24	62.78	13.79	2.077^*^	63.25	13.23	62.89	13.04	60.24	15.84	5.708^**^	1 > 2 > 3
CBF- PI-B													
Neuroticism	20.56	7.52	21.83	7.33	2.934^**^	23.57	7.44	20.26	6.92	19.01	6.79	57.94^**^	1 > 2 > 3
Conscientiousness	33.91	6.25	33.07	6.14	−2.331^*^	33.07	6.21	33.86	5.64	33.10	6.63	2.373	
Agreeableness	34.57	6.62	35.40	5.87	2.221^*^	35.41	6.05	35.31	5.94	34.58	6.31	2.424	
Openness	33.75	6.47	32.84	6.35	−2.420^*^	33.35	6.17	33.26	6.10	32.44	7.07	2.634	
Extraversion	29.93	7.33	29.46	7.08	−1.129	29.55	7.15	29.47	7.26	29.81	7.05	0.244	
Peer relationship													
Peer acceptance	62.34	11.23	62.77	10.12	0.681	61.72	10.52	64.22	10.16	62.62	10.40	7.475^**^	2 > 3 > 1
Peer fear	21.67	7.05	22.66	6.89	2.438^*^	24.06	6.86	21.36	6.71	20.45	6.65	41.259^**^	1 > 2 > 3
Depression (PHQ-9)	5.38	4.82	5.24	4.73	−0.513	5.59	4.87	4.90	4.35	5.13	4.97	2.942	

* *p < 0.05;*

** *p < 0.01*.

### Latent Profile Modeling of Loneliness Profiles

**Figure 1:**
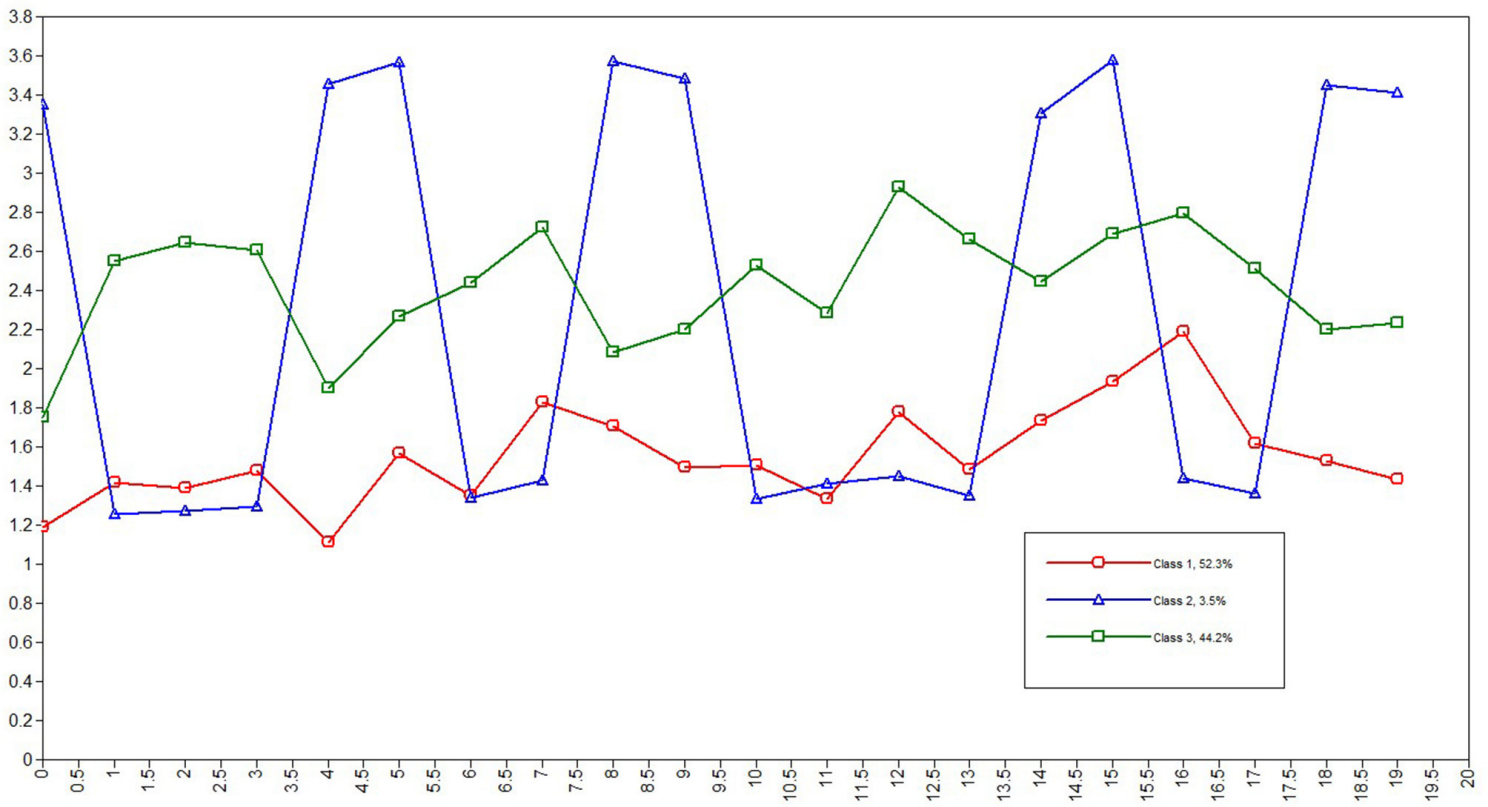
Latent Profile of loneliness.

Three latent profile models were estimated. LPA fit indices for the one- to five-profile models are summarized in Table 3. The three- and four-profile solution had lower AIC, BIC, and aBIC values than both the one- and two-profile solutions, and three-profile solution had significant LMR and BLRT values compared with the four-profile solution. The three-profile solution was selected as the optimal solution for the data. The highest entropy value was 0.949, indicating that the three-profile model provided a clear classification. The three latent profile classes are depicted graphically in Figure 1. The three latent class probabilities were the first class-labeled low loneliness (*n* = 773; 52.3%), the second class-labeled interpersonal sensitivity loneliness (*n* = 52; 3.5%), and the third class-labeled high loneliness (*n* = 653; 44.1%).

**Table 3:** Model fit indices for the latent profile classification analyses (*N* = 1,478).

**Profile**	**k**	**AIC**	**BIC**	**aBIC**	**Entropy**	***p*LMR**	***p*BLRT**	**Class probability**
1 Class	40	73,915.118	74,127.056	73,999.988	–	–	–	1
2 Class	61	66,372.184	66,695.389	66,501.610	0.909	0.0000	0.0000	0.55210/0.44790
**3 Class**	**82**	**63,603.514**	**64,037.986**	**63,777.497**	**0.949**	**0.0004**	**0.0000**	**0.52368/0.03451/0.44181**
4 Class	103	61,214.619	61,760.359	61,433.158	0.933	0.0000	0.0000	0.32070/0.40663/0.03586/0.23681
5 Class	124	60,596.845	61,253.852	60,859.941	0.921	0.0003	0.0000	0.03654/0.28755/0.36739/0.24222/0.06631

*Values in bold indicate the best fitting model. AIC, Akaike Information Criterion; BIC, Bayesian information criterion; aBIC sample size-adjusted Bayesian information criterion; pLMR, p-values for Lo–Mendell–Rubin adjusted likelihood ratio test for K vs. K-1 profiles; pBLRT, p-values for Bootstrapped Likelihood-Ratio Test*.

### Predictors of Different Loneliness Profiles

Two multinomial logistic regressions were performed using the low and interpersonal sensitivity loneliness profiles as the reference groups to determine how variables contributed to each profile. All characteristics of medical students (demographic, stress, life event, coping style, social support, personality, peer relationship, and depression) were entered simultaneously into each of the models. Table 4 presents the association of each predictor variable with the interpersonal sensitivity loneliness profile or the high loneliness profile relative to the low loneliness profile.

**Table 4:** The differences in psychosocial variables between latent profile classes.

**Variables**	**Class#2 vs. Class#1**			**Class#3 vs. Class#1**			**Class#3 vs. Class#2**		
	**Estimate**	**S.E**.	**OR (95%CI)**	**Estimate**	**S.E**.	**OR (95%CI)**	**Estimate**	**S.E**.	**OR (95%CI)**
Age	−0.067	0.224	0.935 (0.603–1.451)	−0.124	0.113	0.883 (0.708–1.102)	0.057	0.222	1.059 (0.685–1.636)
Freshmen	0.133	0.570	1.142 (0.374–3.491)	−0.487	0.194	0.614^*^ (0.420–0.899)	0.620	0.559	1.859 (0.621–5.560)
Sophomore	1.750	0.848	5.755^*^ (1.092–30.328)	0.132	0.234	1.141 (0.721–1.805)	1.619	0.836	5.048^*^ (0.981–25.986)
Junior	2.222	0.822	9.226^**^ (1.842–46.206)	−0.167	0.320	0.846 (0.452–1.584)	2.389	0.813	10.903^**^ (2.216–53.650)
Town	−0.280	0.924	0.756 (0.124–4.623)	0.303	0.235	1.354 (0.854–2.146)	−0.583	0.868	0.558 (0.102–3.060)
Village	−0.083	0.617	0.920 (0.275–3.084)	−0.146	0.272	0.864 (0.507–1.473)	0.063	0.591	1.065 (0.334–3.392)
Single parent family	−0.061	1.029	0.941 (0.125–7.070)	−0.446	0.272	0.64 (0.376–1.091)	0.386	0.958	1.471 (0.225–9.618)
Joint family	0.423	0.541	1.527 (0.529–4.408)	0.045	0.206	1.046 (0.699–1.566)	0.378	0.539	1.459 (0.507–4.197)
Family income									
3,500–5,000 Yuan	−0.302	0.611	0.739 (0.223–2.449)	0.094	0.208	1.099 (0.731–1.651)	−0.396	0.606	0.673 (0.205–2.207)
5,001–8,000 Yuan	0.197	0.651	1.218 (0.340–4.362)	0.191	0.265	1.21 (0.720–2.035)	0.006	0.629	1.006 (0.293–3.452)
>8,000 Yuan	−0.458	0.981	0.633 (0.092–4.327)	0.201	0.323	1.223 (0.649–2.303)	−0.659	1.002	0.517 (0.073–3.687)
Number of siblings									
0	0.126	0.587	1.134 (0.359–3.584)	0.059	0.199	1.061 (0.718–1.567)	0.067	0.571	1.069 (0.349–3.274)
1	0.658	0.641	1.931 (0.550–6.783)	0.092	0.298	1.096 (0.611–1.966)	0.566	0.616	1.761 (0.527–5.891)
Peer acceptance	−0.089	0.039	0.915^*^ (0.848–0.988)	−0.138	0.016	0.871^**^ (0.844–0.899)	0.049	0.039	1.050 (0.973–1.134)
Peer fear	−0.044	0.073	0.957 (0.829–1.104)	0.032	0.023	1.033 (0.987–1.080)	−0.076	0.069	0.927 (0.810–1.061)
PSS	−0.175	0.133	0.839 (0.647–1.089)	0.041	0.045	1.042 (0.954–1.138)	−0.217	0.133	0.805 (0.620–1.045)
Positive coping	−0.019	0.050	0.981 (0.890–1.082)	0.016	0.019	1.016 (0.979–1.055)	−0.034	0.049	0.967 (0.878–1.064)
Negative coping	−0.040	0.065	0.961 (0.846–1.091)	−0.023	0.028	0.977 (0.925–1.032)	−0.017	0.061	0.983 (0.872–1.108)
Family support	0.228	0.246	1.256 (0.776–2.034)	−0.073	0.088	0.93 (0.782–1.105)	0.301	0.242	1.351 (0.841–2.171)
Friend support	0.424	0.228	1.528 (0.977–2.389)	−0.086	0.080	0.918 (0.784–1.073)	0.511	0.226	1.667^*^ (1.070–2.596)
Other support	−0.088	0.039	0.916^*^ (0.848–0.989)	−0.046	0.009	0.955^**^ (0.938–0.972)	−0.042	0.037	0.959 (0.892–1.031)
Aggressive symptoms	−0.066	0.095	0.936 (0.777–1.128)	0.003	0.028	1.003 (0.949–1.060)	−0.069	0.094	0.933 (0.776–1.122)
Avoidance symptoms	−0.201	0.124	0.818 (0.641–1.043)	0.005	0.022	1.005 (0.963–1.049)	−0.206	0.120	0.814 (0.643–1.030)
High arousal symptoms	0.246	0.134	1.279 (0.983–1.663)	0.098	0.038	1.103^**^ (1.024–1.188)	0.147	0.125	1.158 (0.907–1.480)
Neuroticism	0.056	0.067	1.058 (0.927–1.206)	0.056	0.023	1.058^*^ (1.011–1.106)	0.000	0.064	1.000 (0.882–1.134)
Conscientiousness	−0.092	0.042	0.912^*^ (0.840–0.990)	0.006	0.020	1.006 (0.967–1.046)	−0.097	0.041	0.908^*^ (0.837–0.983)
Agreeableness	−0.031	0.046	0.969 (0.886–1.061)	−0.012	0.020	0.988 (0.950–1.028)	−0.019	0.044	0.981 (0.900–1.070)
Openness	0.132	0.057	1.141^*^ (1.020–1.276)	0.028	0.021	1.028 (0.987–1.072)	0.104	0.055	1.110 (0.996–1.236)
Extraversion	−0.004	0.050	0.996 (0.903–1.099)	−0.029	0.020	0.971 (0.934–1.010)	0.025	0.051	1.025 (0.928–1.133)
PHQ-9	0.098	0.064	1.103 (0.973–1.250)	0.027	0.036	1.027 (0.957–1.102)	0.071	0.062	1.074 (0.951–1.212)

* *p < 0.05;*

** *p < 0.01*.

The comparison between the interpersonal sensitivity loneliness profile (Class#2) and the low loneliness profile (Class#1) indicated that sophomore (Est = 2.065; *p* < 0.05) and junior (Est = 2.702; *p* < 0.01) medical students were more likely than freshmen to be classified in the interpersonal sensitivity loneliness profile than low loneliness profile, whereas conscientiousness students (Est = −2.194; *p* < 0.05) were more likely to be classified in the low loneliness profile. Moreover, the students with openness personality predicted membership in the interpersonal sensitivity loneliness profile (Est = 2.303; *p* < 0.05). Students with peer acceptance (Est = −2.266; *p* < 0.05) and other supports (Est = −2.247; *p* < 0.05) predicted membership in the low loneliness profile rather than the interpersonal sensitivity loneliness profile.

The comparison between the high (Class#3) and low loneliness profiles (Class#1) showed that female students than males tended to be classified in the high loneliness profile (Est = −2.515; *p* < 0.05), whereas students with peer acceptance (Est = −8.656; *p* < 0.01) and other supports (Est = −5.058; *p* < 0.01) predicted membership in the low loneliness profile rather than the high loneliness profile. However, students with neuroticism (Est = 2.475; *p* < 0.05) and high arousal symptoms (Est = 2.618; *p* < 0.01) were classified in the high loneliness profile.

The comparison between the interpersonal sensitivity (Class#2) and high loneliness profiles (Class#3) showed that sophomore (Est = 1.937; *p* < 0.05) and junior (Est = 2.939; *p* < 0.05) medical students were more likely than freshmen to be classified in the high loneliness profile. Meanwhile, students with friend's support were more likely to fall into the high loneliness profile (Est = 2.264; *p* < 0.05). However, students with conscientiousness personality were inclined to the interpersonal sensitivity loneliness profile (Est = −2.348; *p* < 0.05). No other significant associations were found.

## Discussion

As the pandemic presented unique psychological challenges for medical students, this study highlighted the loneliness patterns and predictors from post-lockdown to new normal with COVID-19.

### The Differences on Loneliness and Psychosocial Variables Between Gender and Grades

On comparing psychosocial variables, female students had a higher loneliness level than their male counterparts. This may be because females value more on participating in social activities, prefer greater interpersonal connectedness, and are more sensitive to the interpersonal context (22). Therefore, female students may be highly vulnerable to pandemic-related stress and emotional reactions than males. The results corroborated the findings of previous studies that female young adults were at more risk of experiencing social loneliness than males (47). The results showed that female students had higher levels of perceived stress, social support, and peer fear than male students. Perhaps medical students, who pay more attention to epidemic and are more sensitive to it, are easily affected by negative information. A research had shown that female college students were more prone to perceive stress and negative emotions when they were exposed to negative information and threats (48). Previous studies have also confirmed that female students had better interpersonal relationships than males, but their scores on heterosexual communication were lower than those of male students (49–51). This study showed that female students had higher scores in peer fear than males, which might be the influence of the Traditional Chinese Culture. They are likely to maintain a certain distance and interact with males (50). In personality characteristic, female students were more inclined to be more neurotic and agreeable than males. Studies had shown that scores on neuroticism in female students were higher than that in males, and students with high levels of neuroticism were more likely to perceive threats and produce instability mood (52, 53). On the contrary, male students are more responsible and open than females. In the context of Chinese culture, men have to undertake the responsibility of family and are more tolerant toward women in their daily life, which may affect male students' personalities (53).

The results showed that freshmen suffered from a high level of loneliness, perceived stress, life event, negative coping, social support, and peer fear, which are similar to Yang's study (54). The first year of medical school is viewed as an extremely testing time for most students, who are expected to grab a vast amount of information in a competitive learning environment (55). Freshmen also had to leave the support network of family and stay with their classmates or someone they were not familiar with and had high academic pressure, and they poorly adjusted to college life and experienced higher loneliness and stress event, particularly recreational activities were restricted (56).

### Three Latent Profiles of Loneliness Were Identified in Medical Students

Based on the LPA results, three profiles of loneliness were identified. Approximately two in five medical students (44.1%) were categorized in the high loneliness profile. Nearly half of the participants (52.3%) were categorized exclusively in the low loneliness profile. In addition, very few participants (3.5%) were categorized in the interpersonal sensitivity loneliness profile. The “interpersonal sensitivity loneliness” was characterized by the sense of being in interpersonal relationships, such as the items “I feel in tune with the people around me,” “I feel part of a group of friends,” “I have a lot in common with the people around me,” and “I am an outgoing person.” We compared our findings with previous LPA/LCA performed in New Zealand and the United States investigating loneliness heterogeneity in adults. The presence of large low loneliness profile/class was coherent with the previous results (30). On the contrary, the interpersonal sensitivity loneliness profile was only identified in this study. Perhaps, one reason was that different measurements of loneliness were used, and another reason might be the age of participants that varied and peer and interpersonal relationships were most influential for college students (57). Of note, both studies analyzed two public population samples, which were middle aged (mean age, 45.66 years) and aged 18–70 years, respectively. On the other hand, such an increased percentage of high loneliness in this study may be attributed to the social isolation situational risk factors (the campus outside was closed to students from post-lockdown of COVID-19 pandemic to new normal), triggering an increase in the loneliness for college students (58). In addition, campus closure could partly play a role in the development of loneliness among medical students as the school routines, and outside activities changed, particularly for young adults (59). In conclusion, we detected two profiles that were already found in previous studies in adults and identified a novel interpersonal sensitivity loneliness profile, specifically characterized by interpersonal interaction.

### The Predictors for Different Subtype of Loneliness

The results of the multinomial logistic regression analysis showed that sophomore and junior students more likely belonged to interpersonal sensitivity and high loneliness profiles. Prior studies have indicated that students reported more negative effects and physical symptoms during the first term of medical school and reported a greater decrease in positive emotions and perceived peer friendliness (60). However, this result revealed that the elevations of loneliness were likely to be the highest at the end of the first year and started to decline after that was inconsistent with that of a previous study (55). This may be because the freshmen who shifted from tensive study and attention from their parents to more freedom got more support, tolerance, and understanding because as they were new students, they have difficulty in adapting. Zhou's study also found that freshman had more interpersonal disturbance than other grade students (49). Sophomore and junior students relied more on interpersonal relationships. Mostly, they focused on social relationships, social value, acceptance, and self-presentation; being alone may not only include feeling alienated from peers but also the feeling of no social connection (61).

The findings showed that individuals with better peer acceptance and other social supports were classified in the low loneliness profile. Better peer acceptance indicates a good relationship with people. Social support was identified as a protective factor against adversity and stressful conditions (62). A previous research showed that the higher the social support perceived, the better the mental health of college students had, which is mainly related to the enhancing effect of social support on mental health (63). Adequate supports originating from friends, families, and others are crucial to assist an individual in effectively managing stress-provoking situations such as emergency crises and infectious disease outbreaks (64). The other support for medical students came from teachers, campus organization, and students club, which helped the students in coping with the psychological distress and relieving the stress from post-lockdown to new normal.

Personalities influence individual's perceptions of loneliness. Our results indicated that neuroticism students could predict the high loneliness profile. A study pointed that one unit increase in neuroticism was associated with a 1.15 times higher likelihood of loneliness (65). In addition, compared with the low loneliness profile, individuals with openness were more likely to be classified in the interpersonal sensitivity loneliness profile in this study. This finding may explain that openness individuals have larger supportive social networks, with open-minded and diverse interests to engage in new environment, and they pay more attention to the interpersonal interaction (66). Meanwhile, persons with conscientiousness are being responsible, orderly, and dependable to deal with the situation. In this study, students with conscientiousness were intended to have a low loneliness profile where higher conscientiousness would be considered less loneliness. The conscientiousness person would like to help others to deal with distress and obtain high level of social support from others. Wang's study showed that older adults with high conscientiousness had a 24% decreased risk of loneliness (65). In contrast, students with higher friends' support were intended to have a high loneliness profile. Some studies revealed that loneliness was increasingly being seen as a sign of failure to satisfy belongingness needs, which are equal to the lack of interpersonal relationships (67, 68). Belongingness is a mutual social relationship or tie where interpersonal interactions are relatively frequent; it must meet the quantity and quality of the interpersonal relationships (67). Therefore, although the high loneliness profile students had support from several friends, the quality of support was not satisfactory.

## Limitations

In light of these findings, our study had several limitations. First, the participants were selected using the convenience sampling method in one medical university and students with serious mental disorders were excluded; therefore, the generalizability to other students or population is limited. Another limitation was the difference in the number of male and female participants: 1,073 females and 405 males. This could be due to the gender inequality in our university, and the same situation was also observed with the same situational university (69). In addition, 46.5% of the participants were freshmen, and senior students were excluded in this study because they were at a clinical routine; therefore, the inferences should be interpreted with caution. Third, data were collected from a face-on-face Internet survey, which could ensure the authenticity and accuracy of the data. However, as the questionnaires were based on self-report and had relative questions, the recall and response biases could exist. Fourth, it was a cross-sectional study; although the multinomial logistic regressions were used, it is impossible to infer causality between all variables. Longitudinal studies should be conducted to understand the dynamic change of different loneliness profiles and how they relate to psychological variables further.

## Conclusion

Overall, loneliness widely occurred in medical students under the COVID-19–related quarantine, as a special medical reserve group of medical students was substantially affected on psychological and social levels. Our findings identified medical students who were at the highest risk and the predictors for the subtypes of the loneliness profile. These findings provide more robust evidence to best address the psychosocial needs of medical students at this challenge time. The results may offer the college administrators, educators, and mental health professors a new view to identify the high risk of loneliness in medical students on a timely basis and help them deal with hard time and situation that has a profound effect on their future careers and happiness in life. Loneliness-focused interventions targeting the different profiles of loneliness and predictors, campus club activities, volunteer service, peer organizations, and the area of recreation of campus should be established.

## Data Availability Statement

The raw data supporting the conclusions of this article will be made available by the authors, without undue reservation.

## Ethics Statement

The studies involving human participants were reviewed and approved by Ethics Committee of Harbin Medical University. Written informed consent to participate in this study was provided by the participants' legal guardian/next of kin. Written informed consent was obtained from the individual(s), and minor(s)' legal guardian/next of kin, for the publication of any potentially identifiable images or data included in this article.

## Author Contributions

YZ: designed the study. HZ: collected the data and wrote the manuscript. JY: analyzed and interpreted the data. YL and GR: collected and managed the data. LM, YC, and QL: enrolled the participants. All authors contributed to the article and approved the submitted version.

## Conflict of Interest

The authors declare that the research was conducted in the absence of any commercial or financial relationships that could be construed as a potential conflict of interest.
